# Supplementary material for: Hybrid Models and Biological Model Reduction with PyDSTool
Source: PLoS Comput Biol. 2012 Aug 9;8(8):e1002628. doi: 10.1371/journal.pcbi.1002628 (PMC3415397; doi:10.1371/journal.pcbi.1002628)
Supplement: Text S4 — Complete source code for the PyDSTool package (version 0.88.120504). Includes API documentation and help files linking to web pages. This file is identical to the current public release on Sourceforge.net. (ZIP) [file pcbi.1002628.s004.zip › PyDSTool/html/PyDSTool.Toolbox.optimizers.line_search.wolfe_powell_rule.WolfePowellRule-class.html]

xml version="1.0" encoding="ascii"?


PyDSTool.Toolbox.optimizers.line\_search.wolfe\_powell\_rule.WolfePowellRule


| Home | Trees | Indices | Help | PyDSTool |
| --- | --- | --- | --- | --- |

Package PyDSTool ::
Package Toolbox ::
Package optimizers ::
Package line\_search ::
Module wolfe\_powell\_rule ::
Class WolfePowellRule

[hide private]

[frames] | no frames]

# Class WolfePowellRule

source code

```
object --+
         |
        WolfePowellRule
```

---

The standard Wolfe-Powell rule for a inexact line search


|  |  |
| --- | --- |
| Instance Methods | [hide private] |

|  |  |
| --- | --- |
| \_\_init\_\_(self, alpha=1.0, rho=0.1, sigma=0.4, alpha\_min=0.0, alpha\_max=1.0, alpha\_limit=0.1, \*\*kwargs)  Initializes the search Parameters : | source code |

|  |  |
| --- | --- |
| \_\_call\_\_(self, origin, function, state, \*\*kwargs)  Tries to find an acceptable candidate | source code |

**Inherited from `object`**:
`__delattr__`,
`__getattribute__`,
`__hash__`,
`__new__`,
`__reduce__`,
`__reduce_ex__`,
`__repr__`,
`__setattr__`,
`__str__`


|  |  |
| --- | --- |
| Properties | [hide private] |

**Inherited from `object`**:
`__class__`


|  |  |
| --- | --- |
| Method Details | [hide private] |

|  |  |
| --- | --- |
| \_\_init\_\_(self, alpha=1.0, rho=0.1, sigma=0.4, alpha\_min=0.0, alpha\_max=1.0, alpha\_limit=0.1, \*\*kwargs)  *(Constructor)* | source code |

Initializes the search Parameters :

- alpha is the first step size that will be tried (1.)
- rho is the rhos acceptation factor (0.1)
- sigma is the factor for the Wolfe-Powell rule (0.4)
- alpha\_min is the inf limit of the search interval (0.)
- alpha\_max is the max limit of the search interval (1.)
- alpha\_limit is a factor so that the estimated alpha is not near the
  limts of the tested bracket, leading to a divergence in the algorithm
  (alpha\_limit = 0.1)

Those parameters should be tweaked depending on the function to
optimize

Overrides: object.\_\_init\_\_

  


| Home | Trees | Indices | Help | PyDSTool |
| --- | --- | --- | --- | --- |

|  |  |
| --- | --- |
| Generated by Epydoc 3.0.1 on Fri May 4 15:24:09 2012 | http://epydoc.sourceforge.net |
